# A comparison of traditional and novel metrics to quantify resistance training

**DOI:** 10.1038/s41598-017-05953-2

**Published:** 2017-07-17

**Authors:** Kieran J. Marston, Jeremiah J. Peiffer, Michael J. Newton, Brendan R. Scott

**Affiliations:** School of Psychology and Exercise Science, Murdoch University, Perth, Western Australia Australia

## Abstract

Common estimates of external training intensity for resistance exercise do not incorporate inter-set recovery duration, and might not reflect the overall demands of training. This study aimed to assess novel metrics of exercise density (ED) during resistance exercise, and how these related to a physiological marker of internal training intensity as well as traditional measures of external training intensity and volume. Thirteen males and seven females performed two bouts of resistance exercise focused on developing strength (5 sets of 5 repetitions with 5-repetition maximum; 180 s recovery) and hypertrophy (3 sets of 10 repetitions with 10-repetition maximum; 60 s recovery). Blood lactate concentration was measured to quantify internal training intensity. Specific metrics of external volume (mechanical work, volume load and total repetitions) and intensity (average weight lifted and ED) were calculated. Despite lower average weights and no difference in mechanical work or volume load, blood lactate was greater following hypertrophy compared with the strength condition. This finding was consistent with higher measures of ED in the hypertrophy compared with the strength condition. Greater ED during hypertrophy resistance exercise, along with the significant association with changes in blood lactate, indicates that ED metrics are reflective of the sessional intensity for resistance exercise.

## Introduction

Excessive training intensity or volume can result in chronic exercise stress, particularly if coupled with additional stressors such as increased training frequency or regular travel^1–4^. Under such circumstances, athletes can experience a decline in the quality of subsequent training bouts or sports performance^1, 5^. If the training stress is not attenuated, these athletes can experience suppression of the immune system, decreased muscle glycogen storage, chronic muscle damage, neuroendocrine fluctuations and a disrupted psychological state^1–4^. Careful monitoring of the training dose is essential to ensuring an athlete can perform at their highest level. Monitoring training intensity and training load (the product of intensity and volume) for endurance^6, 7^ and team sport^8, 9^ athletes has received considerable attention. However, strategies to quantify resistance training, a common component of most athletes’ training programs, have not been extensively explored^10^. This is surprising, considering that lifting the same relative weight for equivalent total volume, yet manipulating acute variables (e.g. recovery periods, repetition velocity and lifting tempo), can produce a vastly different training stimulus^10^.

To date, calculating the mechanical work completed during a resistance training session is the most valid method for quantifying external resistance training volume^11^. However, this process is time-consuming and requires specialized equipment, limiting its practical application^12^. Alternatively, metrics such as the repetition method (providing total repetitions) and volume load (VL) are used to quantify training volume, though these methods are also inherently limited. The repetition method involves the summation of repetitions performed during a training session or cycle^12^ and while easy to implement and interpret, this system fails to account for weight lifted during each repetition. Instead, VL is determined by the product of repetitions performed and the mass lifted to provide an absolute value in kilograms (sometimes termed tonnage)^11, 13^, and can provide an estimate of the mechanical work completed during resistance training as well as the associated physiological stress^11, 14^. However, an individual training at 70% of 1 repetition maximum (RM) will have an identical VL whether that session is performed for 10 sets of 3 repetitions or 3 sets of 10 repetitions, despite experiencing a vastly different physiological stimulus^10^. Therefore, additional metrics are necessary to differentiate the overall intensity of training bouts.

The intensity of resistance training is often represented as the weight lifted relative to an individual’s maximal strength for a single repetition of that exercise (i.e. 70% of 1-repetition maximum [1 RM])^15^. While this is a simple assessment for the intensity of an individual repetition, it does not adequately describe the intensity of a training session when considering manipulation of additional acute exercise variables. Alternatively, the Training Intensity (TI) metric developed by Stone *et al*.^13^ quantifies the intensity of a training session by calculating the average weight lifted throughout a session from the division of VL by the total repetitions performed. Although these methods indicate the intensity of the weight lifted for a single repetition and a series of repetitions, respectively, the ‘true intensity’ of a training session is far more complex involving both mechanical (external) and metabolic (internal) work^16^. The ‘true intensity’ of an exercise session is affected by varying session designs (sets and repetitions)^16^, the load lifted^17, 18^, inter-set recovery durations^19, 20^ and repetition velocity^20^. To illustrate, recovery duration influences muscle force production^21, 22^ and hormonal responses^23^. Shorter inter-set recovery durations during resistance exercise are also associated with elevations in the blood lactate concentration^24^ while longer periods of recovery result in greater passive metabolite clearance^25^.

It is likely that the sessional intensity of resistance training is a cumulative measure that is reliant on the interplay between the volume, intensity and recovery associated with each individual set. However, to the authors’ knowledge there is no objective method of quantifying sessional resistance training intensity that accounts for the influence of varied inter-set rest periods^13, 26^. Given the outlined limitations with common metrics of external training volume and intensity, we sought to create an objective metric to quantify sessional training intensity. We expanded on the term “density” described by Bompa *et al*.^27^ to create novel metrics of “exercise density” (ED) derived from the division of mechanical work and VL by sessional recovery time measured in seconds. Through the use of two distinctively different, yet work and volume matched resistance training protocols, we sought to examine whether the novel metrics were; 1) able to differentiate between these protocols, and 2) provide an estimate of sessional intensity as measured by change in blood lactate concentration, a commonly used measure of glycolytic metabolism and exercise demand^24^. We hypothesized that although traditional strength training is performed using higher intensities for each repetition (i.e. 85–95% of 1 RM), traditional hypertrophy training would result in a greater sessional intensity and that this finding would be supported by the novel ED metrics and changes in blood lactate.

## Methods

### Participants

Thirteen males (age: 25.0 ± 1.4 years) and seven females (age: 23.4 ± 1.4 years) with novice to intermediate resistance training experience volunteered for participation in this study. At the time of the study only five of the 20 participants were involved in structured resistance training greater than twice a week. Participants were considered low risk for moderate to intense exercise as per an established screening questionnaire^28^. Participants were informed of the study’s aims and provided written informed consent. Ethical approval for this study was provided by the Murdoch University Human Research Ethics Committee. Research was carried out in accordance with the institutional guidelines and regulations.

### Design

Participants were required to complete five exercise sessions. The first session was used to familiarize participants with the procedures and equipment used in the study. During this session, lifting technique was assessed for each of the seven resistance exercises (bench press, seated row, leg press, lat pull-down, military press, leg extension, and arm curl) and visual and verbal instructions were provided to ensure correct form. The following two sessions were completed in a randomized order and were used to assess the 5- and 10 RM for each of these exercises. Participants then completed the remaining two resistance exercise protocols in a randomized and counterbalanced order. These protocols were designed provide a similar level of work, but different training stimuli, to investigate the ability of different metrics to quantify various resistance training protocols. Total mechanical work was theoretically matched *a priori* using the %1 RM-Repetition Relationship described previously^16^. Earlier findings indicate that untrained individuals are able to lift fewer repetitions at a given percentage of 1 RM when compared to trained individuals^29^; thus, a conservative estimate (i.e. 70% of 1 RM to approximate 10 RM, 85% of 1 RM to approximate 5 RM) was used to theoretically match mechanical work. All exercise sessions were conducted with four to ten days of recovery between sessions.

### Definition of Terms

Inconsistent use of resistance training terminology within the field of strength and conditioning has added to the difficulty in quantifying resistance training variables^15, 30^. Instead of recognizing a uniform meaning, terms such as intensity, volume and load are routinely used to define a variety of resistance training factors. In this paper, the following definitions of key terms will be used; (1) mechanical work: the product of an applied force multiplied by the corresponding displacement in the direction of that force^31^, (2) metabolic work: the processing of chemical substrates (i.e. glucose and fat metabolism) for energy production and use by the body^32^, (3) VL: the product of weight lifted and repetitions performed^10^, (4) weight intensity: the weight lifted during training reported relative to maximal strength in a particular exercise (% 1 RM) or as the average weight lifted across a session (TI)^12, 13^, (5) internal training intensity: metabolic work as measured by changes in blood lactate concentration, (6) sessional intensity: the effort or stress associated with performing an entire training session^10^, and 7) exercise density: the mechanical work or VL of a training session reported relative to the summed inter-set recovery periods (expressed as J∙s^−1^ or kg∙s^−1^, respectively).

### Procedures

#### Maximal Strength Testing

Maximal strength was assessed via 5- and 10 RM testing conducted on separate days in a randomized order. Seven resistance exercises (bench press, seated row, leg press, lat pull-down, military press, leg extension, and arm curl) were performed in order of mention for both the maximal testing and experimental sessions. Prior to 5 RM assessment, participants performed a 5-min general warm-up on a rowing ergometer at self-selected intensity, following by a specific warm-up of each resistance exercise (light weight repetitions). Participants were instructed to lift a resistance pre-selected by the researcher with the aim of performing five repetitions with correct technique and without requiring assistance. If the set was successful and the participant was capable of lifting the additional weight, the resistance was increased by small increments until the participant could not complete five repetitions with correct technique. Participants rested for periods of 120–240 s between RM attempts to ensure adequate recovery as per testing guidelines^16^. During each RM attempt, the displacement of the weight stack, sled or bar was measured using a tape measure for *post hoc* calculation of mechanical work. This technique was adopted from previous research, whereby displacement was measured over several repetitions during the set to create a mean displacement^33^. Throughout repetitions of the arm curl, a goniometer was placed over the lateral epicondyle of the humerus to measure the individual degree of flexion. Furthermore, the length of the moment arm during the arm curl was measured, using a tape measure, from lateral epicondyle of the humerus to mid palm where the dumbbell was held. The 10 RM assessment was completed using an identical methodology to the 5 RM, with the exception that the maximum mass that could be lifted with correct technique for ten repetitions was determined. All RM assessments were conducted by a single rater throughout the study.

#### Exercise Protocols

All experimental trials commenced with a 5-min general warm-up on a rowing ergometer at a self-selected intensity, followed by a specific warm-up for each exercise to be completed using light weights (single set of ten repetitions at 50% of 10 RM). During the strength training session, participants completed 5 sets of 5 repetitions using 5 RM weight, with 180 s of passive inter-set recovery. The hypertrophy training session consisted of 3 sets of 10 repetitions with 10 RM weight and 60 s inter-set recovery. All testing was completed at the same time of day to account for circadian rhythm, and participants were asked to abstain from strenuous exercise 24 hours prior to the testing sessions. Exercises were performed in the same order as during the RM testing.

### Measure of Internal Training Intensity

Internal training intensity was quantified by measuring blood lactate before and immediately after completion of the exercise session. Blood lactate was measured in duplicate from a 0.7 µl capillary blood samples obtained from a fingertip which was immediately analyzed using a hand-held lactate analyzer (Lactate Plus, Nova Biomedical^®^, USA). The mean value of the duplicate measures was used for analysis to reduce measurement error. The test-retest reliability of a single rater demonstrated an intraclass correlation coefficient (ICC) indicative of strong agreement and reliability (ICC = 0.99; 95% confidence interval, 0.94 to 0.99).

### External Volume and Intensity Calculations

The volume and intensity of exercise during each session were calculated using the equations listed in Table 1. Mechanical work for exercises where the displacement of the mass was measured vertically (bench press, military press, leg extension, seated row, lat pull-down) was calculated using equation (1); where *W* is mechanical work (J), f is force (N), and d is displacement (m). Equation (2) was used to calculate mechanical work for the 45° incline leg press exercise and uses the standard mechanical work equation along with cosine and an angle of 45°. Accounting for rotation about the elbow joint, equation (3) calculates mechanical work for the arm curl using torque (N∙m) and θ as range of motion (radians). The work for each repetition performed was summed to calculate mechanical work for each exercise and the session. Total sessional VL and total repetitions were calculated using equations (4) and (5), respectively, to provide simple indications of training volume. Intensity was calculated as the average intensity of the weight lifted via the TI equation (6), while ED metrics were calculated to indicate sessional intensity. Measures of ED were calculated two ways; a) derived from mechanical work (ED_*W*_) by dividing mechanical work by summed inter-set recovery (equation 7), and b) derived from VL (ED_VL_) by dividing VL by summed inter-set recovery (equation 8).

**Table 1:** Equations used to calculate external volume and intensity.

Equation No.	External Measure	Equation
**Measures of External Volume**		
1	Mechanical Work	*W* = f ∙ d
2	Mechanical Work (Leg Press)	*W* = (f ∙ cosine ∙ 45) ∙ d
3	Mechanical Work (Arm Curl)	*W* = t ∙ θ
4	Volume Load	VL = m ∙ repetitions
5	Total Repetitions	TR = sets ∙ repetitions
**Measures of Exercise Intensity**		
6	Training Intensity	TI = VL/repetitions
7	Exercise Density (Work)	ED_*W*_ = *W*/s
8	Exercise Density (Volume Load)	ED_VL_ = VL/s

*Note*. ***W*** **= **mechanical work, **f = **force, **d = **displacement, **t = **torque, **θ = **theta (angle in radians), **VL = **volume load, **m = **mass, **TR = **total repetitions, **TI** = training intensity, **ED = **exercise density, **s = **seconds.

### Statistical Analyses

Differences in lactate between exercise protocols (strength and hypertrophy) over time (pre- and post-exercise) were assessed using a linear mixed model. Significant main effects or interactions were assessed using the LSD *post hoc* test. Differences within each remaining variable were assessed using a dependent *t*-test. Effect sizes (ES; Hedge’s g) estimated the magnitude of difference in mechanical work, VL, blood lactate and ED between conditions. Effect sizes were calculated using mean difference and mean pooled standard deviations and categorized as small (0.20–0.49), moderate (0.50–0.79) or large (≥0.80)^34^. Relative changes in blood lactate pre- to post-resistance exercise (merged hypertrophy and strength data) were correlated (Pearson’s *r*) with mechanical work, VL, total repetitions, ED_*W*_, ED_VL_ and TI. Correlation sizes were interpreted as high (0.70 to 0.90), moderate (0.50 to 0.70) or low (0.30 to 0.50)^35^. Examination of data revealed no outliers prior to correlation using established methods of outlier labeling^36^, and a subsequent Fisher’s transformation allowed the comparison of correlations using *z*-scores. Mixed modelling, correlation coefficients, dependent *t*-test and *post hoc* statistical analyses were conducted using SPSS analysis software (Version 22, IBM^®^, U.S.A) with the level of significance set at *p* ≤ 0.05. *Post hoc* analysis of achieved statistical power was conducted using G*Power (Version 3.1.9.2, Germany). For our primary measure of blood lactate, we calculated a *post hoc* achieved statistical power of 0.99 for our sample size of 20. All data are presented as mean ± SD unless otherwise noted.

## Results

**Figure 1:**
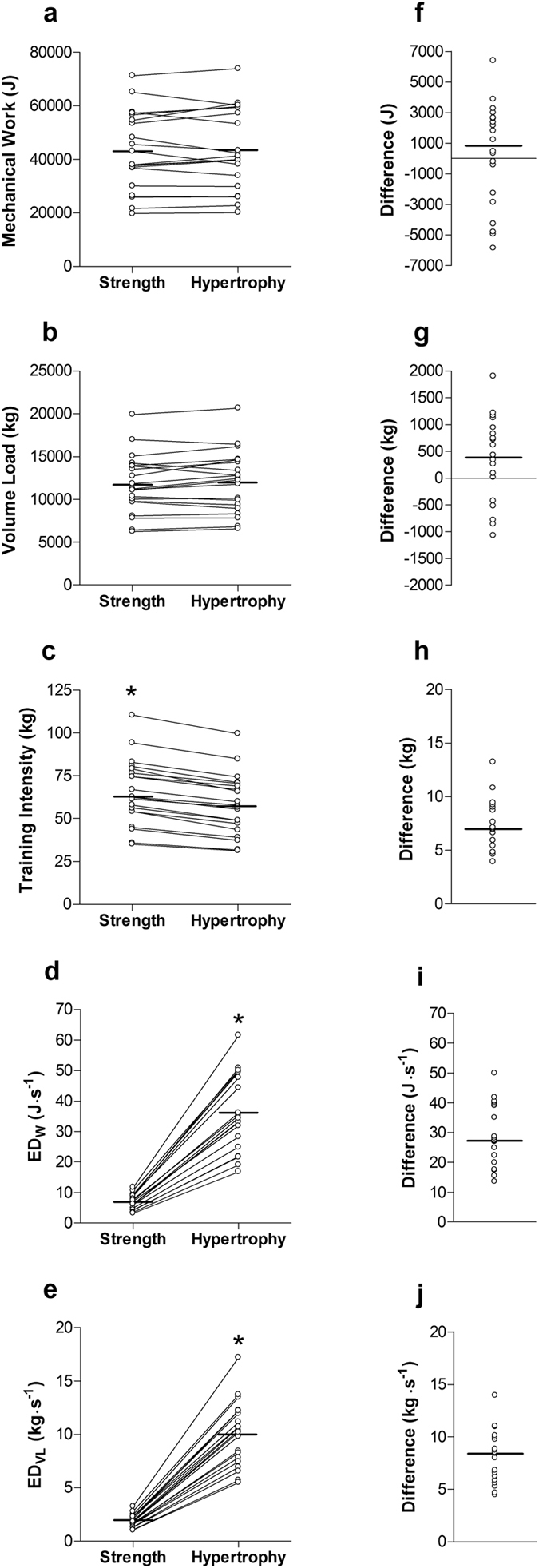
Paired comparisons between strength and hypertrophy conditions about the median (−) in (**a**) mechanical work, (**b**) Volume Load, (**c**) TI, (**d**) ED_*W*_ and (**e**) ED_VL_. Pairwise differences in (**f**) mechanical work, (**g**) Volume load, (**h**) TI, (**i**) ED_*W*_ and (**j**) ED_VL_. *Difference significant at *p* < 0.01.

Across all exercises, participants failed to complete a total of 1.7 ± 2.6 (<1%) repetitions during strength and 11.1 ± 11.7 (4.8%) repetitions during the hypertrophy conditions. By design, a significant difference (*p* < 0.01; ES = 3.53) was observed in total repetitions, with participants completing a greater amount of repetitions during the hypertrophy (228.9 ± 11.7 repetitions) when compared with the strength condition (198.3 ± 2.6 repetitions). No differences (*p* = 0.78; ES = 0.01) in mechanical work were observed between strength and hypertrophy conditions (Fig. 1a). Mean VL (Fig. 1b) between the strength and hypertrophy conditions was not different (*p* = 0.10; ES = 0.09). Mean TI was greater (*p* < 0.01, ES = 0.40) in the strength when compared with the hypertrophy condition (Fig. 1c).

**Figure 2:**
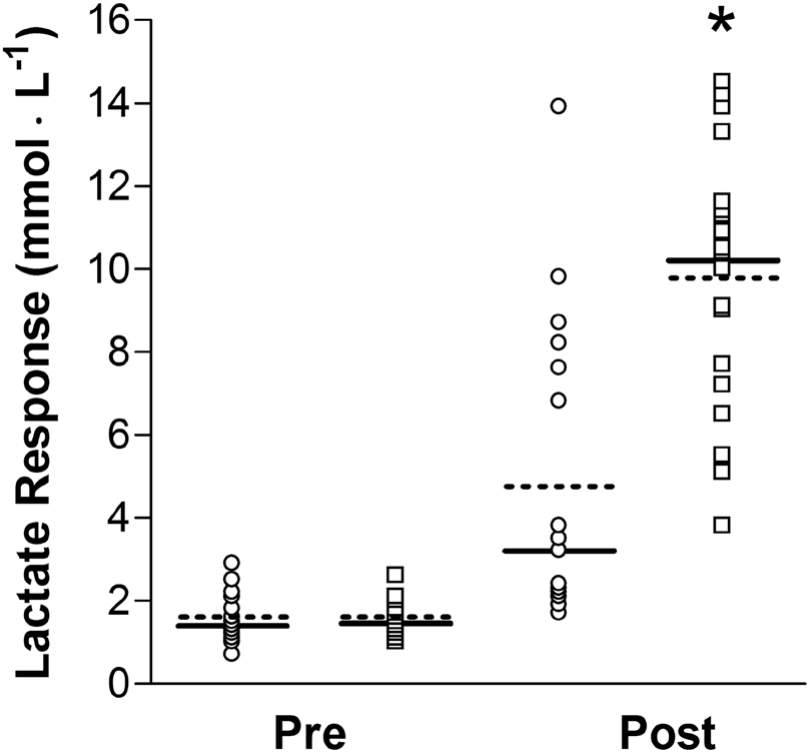
Mean (- - -) and median (—) blood lactate responses from pre- to post-exercise in strength (○) and hypertrophy (□) conditions. *Post-exercise blood lactate levels greater in the hypertrophy than the strength condition (*p* < 0.01).

**Figure 3:**
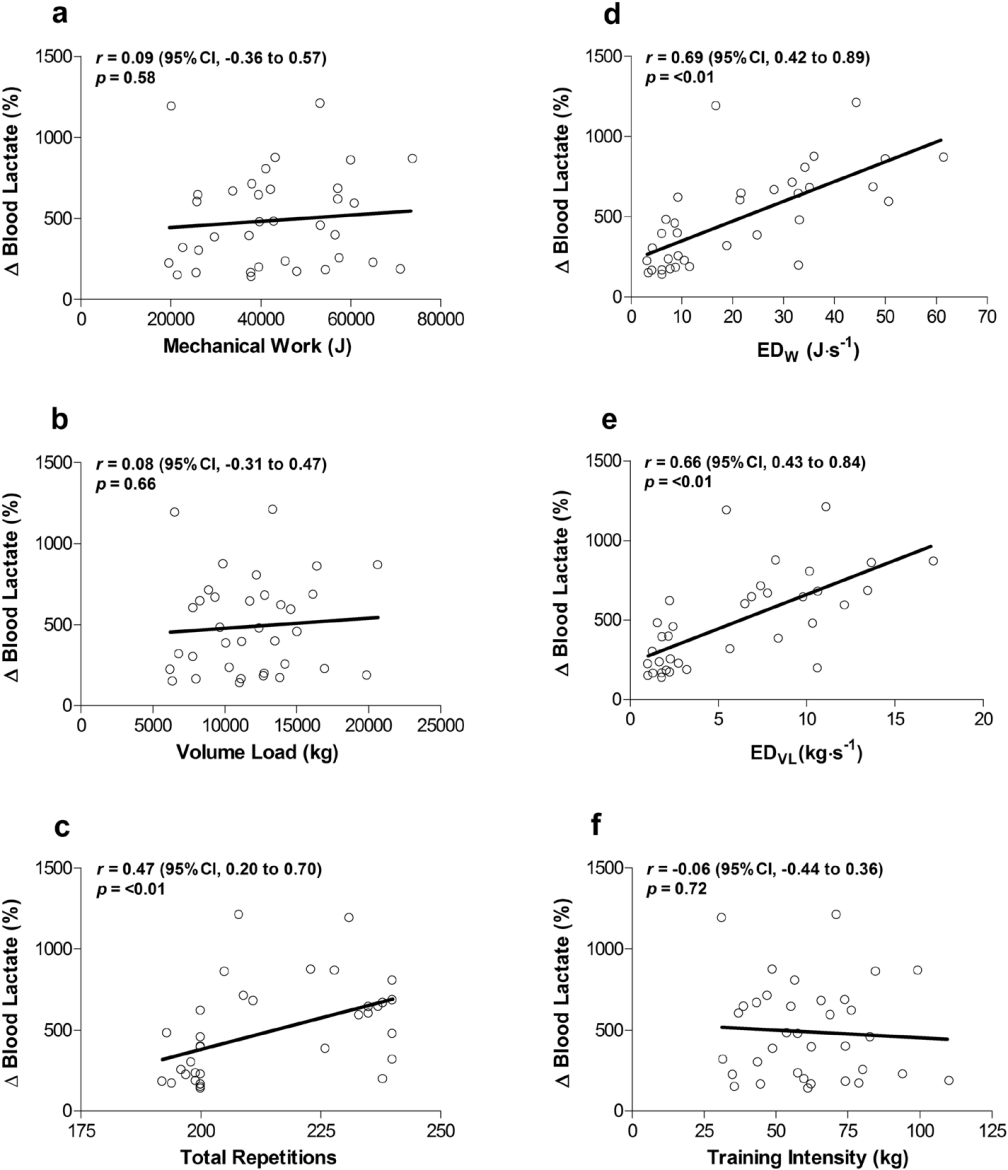
Pearson correlations between relative change in blood lactate concentration following resistance exercise and (**a**) mechanical work, (**b**) Volume Load, (**c**) Total Repetitions, (**d**) ED_*W*_, (**e**) ED_VL_ and (**f**) TI. *Correlation is significant at *p* < 0.01.

Greater ED_*W*_ was observed during the hypertrophy when compared with the strength condition (Fig. 1d; *p* < 0.01; ES = 3.18). Similarly, ED_VL_ was significantly greater following hypertrophy when compared with the strength condition (Fig. 1e; *p* < 0.01; ES = 3.68). Blood lactate concentration demonstrated no pre-exercise difference between conditions (Fig. 2; *p* = 0.99). Blood lactate variability (coefficient of variation) was determined to be 15.7% (95% confidence interval [CI], 11.6 to 24.0%) at baseline, and 17.6% (95% CI, 13.0 to 27.1%) immediately after exercise. Post-exercise measures of blood lactate were significantly greater following hypertrophy when compared with the strength condition (*p* < 0.01; ES = 1.49). Relative changes in blood lactate were not correlated with volume metrics mechanical work (Fig. 3a) or VL (Fig. 3b), and moderately correlated with total repetitions (Fig. 3c). However, they were moderately correlated with sessional intensity metrics ED_*W*_ (Fig. 3d) and ED_VL_ (Fig. 3e). Relative changes in blood lactate were not correlated with intensity metric TI (Fig. 3f). A Fisher’s *r*-*z* transformation revealed no significant differences between the correlations for ED_*W*_ and ED_VL_ (*p* = 0.82, *z* = 0.22), ED_VL_ and total repetitions (*p* = 0.26, *z* = 1.13) or ED_*W*_ and total repetitions (*p* = 0.18, *z* = 1.35).

## Discussion

The purpose of this study was to compare novel methods for determining sessional intensity during resistance exercise with a marker of metabolic stress to highlight the most appropriate metrics for quantifying various types of resistance training. The main findings of this study were; (1) both ED metrics indicated greater sessional intensity following the hypertrophy compared with the strength condition, (2) increases in blood lactate were only significantly correlated with ED metrics and total repetitions, and (3) common metrics of resistance training intensity (TI) and volume (VL and mechanical work) did not differentiate between protocols in acordance with blood lactate or ED.

Blood lactate concentration is often used to reflect the metabolic stress and internal intensity associated with an exercise session^22, 37^. In accordance with previous research^24, 37^, blood lactate concentration was significantly greater following hypertrophy when compared with strength resistance exercise, even when matched for mechanical work and VL. The difference in the lactate response (Fig. 2) was likely influenced by the manipulation of inter-set recovery periods, as shorter periods of inter-set recovery are associated with decreased clearance of blood lactate from the muscle^25^, which accumulates with each performed repetition^38^. We cannot disregard that other variables, that were purposely manipulated (i.e. number of repetitions per set), also contributed to the greater lactate response observed in the hypertrophy condition. While this may have resulted in an increased lactate response compared with the strength condition during each set, the use of shorter recovery periods during the hypertrophy condition would have resulted in a continued elevation of lactate throughout the training session, ultimately indicating a greater level of metabolic stress^37^.

Previous methods of quantifying external training volume and intensity (i.e. repetition method, VL and TI) have quantified total repetitions or the weight lifted with limited consideration of other training variables such as inter-set recovery^10^. The simplicity of the repetition method, VL and TI makes these attractive strategies for monitoring a training dose. However, they do not account for all training variables that influence the physical demands or physiological strain of an exercise session. By design, greater total repetitions were performed in the hypertrophy when compared with the strength condition, whereas TI was significantly greater during the strength when compared with the hypertrophy condition. It is important to note that the differences in the total repetitions and TI are a reflection of our controlled protocol design to match mechanical work by controlling sets, repetitions and relative weight (5 RM and 10 RM). TI can provide interesting information for monitoring day-to-day training demands if training variables including inter-set recovery duration and repetition velocity remain constant^12^. However, as this method only accounts for weight lifted and total repetitions, TI appears problematic for quantifying sessional training intensity when additional variables are manipulated.

Unlike the TI method, previous research indicates that measures of VL are associated with physiological responses during resistance exercise^14^. This would suggest that as VL was matched between exercise protocols in this study, the strength and hypertrophy exercise sessions should have resulted in a similar physiological response; yet, as observed with the lactate response, this was not the case. Since the metabolic stress of a resistance training session is dependent on the weight lifted, the number of sets, inter-set recovery duration, repetitions at a given resistance and repetition velocity, it is unlikely VL can fully reflect metabolic stress. In accordance with our hypothesis, ED metrics were able to differentiate between hypertrophy and strength protocols where VL and mechanical work could not. Significantly greater ED_*W*_ and ED_VL_ were calculated following the hypertrophy protocol when compared with the strength protocol. Considering that the ‘true intensity’ of an exercise session is associated with both mechanical and metabolic output^16^, by not objectively quantifying sessional intensity, significant metabolic-specific training stresses may be unaccounted for.

Interestingly, relative change in blood lactate concentration from pre- to post-exercise was moderately correlated with total repetitions, ED_*W*_, and ED_VL_, despite no significant correlation with the traditional methods of mechanical work, VL or TI. These findings further support our hypothesis that ED metrics better represent the metabolic stress of a resistance training session when compared with more traditional methods. The moderate correlation observed with total repetitions is not surprising as lactate accumulation occurs with each repetition^38^; thus, the higher number of total repetitions present in the hypertrophy when compared with the strength condition would have led to this finding.

It should be acknowledged that ED metrics might not be applicable in all formats of resistance exercise, particularly when training for muscular power. Repetition velocity is not considered in the ED metrics, and they therefore might not be appropriate for quantifying the sessional intensity associated with power training. Nevertheless, the same argument could be made for the traditional metrics of VL, TI and total repetitions, and future studies should investigate ED metrics which incorporate repetition velocity to enhance monitoring processes for power training. Additional research is also needed to increase understanding of ED in relation to session-rating of perceived exertion, as this technique is commonly implemented in resistance training and has been shown to reflect the overall stress of a session^39^. This would assist in confirming the relationships observed between ED metrics and internal training intensity, measured via blood lactate in this study, considering that plasma volume may be altered differently in response to hypertrophy and strength training protocols. While the differences in plasma shifts between 5- and 10 RM protocols are small^37^, it may influence the accuracy of blood lactate measurements. Finally, due to the substantial differences between the structure of the two exercise protocols investigated, we are unable to confirm whether the relationship between the ED metrics and response in lactate would continue in the instance 5- and 10 RM training structures are matched for inter-set recovery duration. However, these protocols were chosen to represent ecologically valid and peer-reviewed training methods^23^ that allowed us to instead examine the effect of inter-set recovery on sessional training intensity in two viable training methods from a practical perspective.

In conclusion, standard methods for quantifying resistance training provide limited insight into the sessional training intensity of an exercise session for untrained individuals. The ED metrics demonstrated moderate correlations with a marker of metabolic stress in this population, while traditional measures of resistance training volume and intensity did not. Based on these findings, ED metrics should be considered by individuals aiming to quantify sessional intensity of resistance training bouts.

## References

[1] Fry RW, Morton AR, Keast D (1991). Overtraining in athletes. An update. Sports Med.

[2] Angeli A, Minetto M, Dovio A, Paccotti P (2004). The overtraining syndrome in athletes: A stress-related disorder. J. Endocrinol. Invest..

[3] Foster C (1998). Monitoring training in athletes with reference to overtraining syndrome. Med. Sci. Sports Exerc..

[4] Mackinnon LT (2000). Overtraining effects on immunity and performance in athletes. Immunol. Cell Biol..

[5] Fry AC, Kraemer WJ (1997). Resistance exercise overtraining and overreaching. Neuroendocrine responses. Sports Med.

[6] Roos L, Taube W, Brandt M, Heyer L, Wyss T (2013). Monitoring of daily training load and training load responses in endurance sports: What do coaches want?. Schweizerische Zeitschrift fur Sportmedizin und Sporttraumatologie.

[7] Esteve-Lanao J, Foster C, Seiler S, Lucia A (2007). Impact of training intensity distribution on performance in endurance athletes. J. Strength Cond. Res..

[8] Coutts AJ, Rampinini E, Marcora SM, Castagna C, Impellizzeri FM (2009). Heart rate and blood lactate correlates of perceived exertion during small-sided soccer games. J. Sci. Med. Sport.

[9] Stagno KM, Thatcher R, van Someren KA (2007). A modified TRIMP to quantify the in-season training load of team sport players. J. Sports Sci..

[10] Scott BR, Duthie GM, Thornton HR, Dascombe BJ (2016). Training Monitoring for Resistance Exercise: Theory and Applications. Sports Med.

[11] McBride JM (2009). Comparison of methods to quantify volume during resistance exercise. J. Strength Cond. Res..

[12] Haff GG (2010). Quantifying workloads in resistance training: a brief review. Prof. Strength Cond.

[13] Stone M (1999). Periodization: Effects Of Manipulating Volume And Intensity. Part 1. Strength Cond. J.

[14] Genner KM, Weston M (2014). A comparison of workload quantification methods in relation to physiological responses to resistance exercise. J. Strength Cond. Res..

[15] Fry AC (2004). The role of resistance exercise intensity on muscle fibre adaptations. Sports Med.

[16] Haff, G. G. & Triplett, N. T. *Essentials of Strength Training and Conditioning* (4th ed.) (Human Kinetics, 2016).

[17] Gearhart RF (2002). Ratings of perceived exertion in active muscle during high-intensity and low-intensity resistance exercise. J. Strength Cond. Res..

[18] Lagally KM, Robertson RJ, Gallagher KI, Gearhart R, Goss FL (2002). Ratings of perceived exertion during low- and high-intensity resistance exercise by young adults. Percept. Mot. Skills.

[19] Willardson JM, Burkett LN (2005). A comparison of 3 different rest intervals on the exercise volume completed during a workout. J. Strength Cond. Res..

[20] Kraemer WJ, Ratamess NA (2004). Fundamentals of Resistance Training: Progression and Exercise Prescription. Med. Sci. Sports Exerc..

[21] Pincivero DM, Gear WS, Moyna NM, Robertson RJ (1999). The effects of rest interval on quadriceps torque and perceived exertion in healthy males. J. Sports Med. Phys. Fitness.

[22] Hardee JP (2012). Effect of inter-repetition rest on ratings of perceived exertion during multiple sets of the power clean. Eur. J. Appl. Physiol..

[23] Kraemer WJ (1990). Hormonal and growth factor responses to heavy resistance exercise protocols. J. Appl. Physiol..

[24] Kraemer WJ, Noble BJ, Clark MJ, Culver BW (1987). Physiologic responses to heavy-resistance exercise with very short rest periods. Int. J. Sports Med..

[25] Ratel S, Bedu M, Hennegrave A, Doré E, Duché P (2002). Effects of age and recovery duration on peak power output during repeated cycling sprints. Int. J. Sports Med..

[26] Stone, M. H., Stone, M., Sands, W. A. & Sands, B. *Principles and practice of resistance training* (Human Kinetics, 2007).

[27] Bompa, T. & Haff, G. G. *Periodization* (5th ed.) (Human Kinetics, 2009).

[28] Coombes, J. & Skinner, T. *ESSA*’*s Student Manual for Health*, *Exercise and Sport Assessment* (Elsevier Health Sciences, 2014).

[29] Hoeger WWK, Hopkins DR, Barette SL, Hale DF (1990). Relationship between Repetitions and Selected percentages of One Repetition Maximum: A Comparison between Untrained and Trained Males and Females. J. Strength Cond. Res..

[30] Knuttgen HG, Kraemer WJ (1987). Terminology and measurement in exercise performance. J. Strength Cond. Res..

[31] Radin, S. H. & Folk, R. T. *Physics for Scientists and Engineers* (Prentice-Hall, 1982).

[32] Garhammer J (1993). A Review of Power Output Studies of Olympic and Powerlifting: Methodology, Performance Prediction, and Evaluation Tests. J. Strength Cond. Res..

[33] Blazevich AJ, Jenkins DG (2002). Effect of the movement speed of resistance training exercises on sprint and strength performance in concurrently training elite junior sprinters. J. Sports Sci..

[34] Cohen, J. *Statistical Power Analysis for the Behavioral Sciences* (2nd ed.) (Lawrence Erlbaum, 1988).

[35] Hinkle, D. E., Wiersma, W. & Jurs, S. G. *Applied statistics for the behavioral sciences* (5th ed.) (Houghton Mifflin, 2003).

[36] Hoaglin DC, Iglewicz B (1987). Fine-Tuning Some Resistant Rules for Outlier Labeling. J. Am. Stat. Assoc.

[37] Kraemer WJ (1993). Effects of different heavy-resistance exercise protocols on plasma β- endorphin concentrations. J. Appl. Physiol..

[38] Denton J, Cronin JB (2006). Kinematic, kinetic, and blood lactate profiles of continuous and intraset rest loading schemes. J. Strength Cond. Res..

[39] McGuigan MR, Egan AD, Foster C (2004). Salivary cortisol responses and perceived exertion during high intensity and low intensity bouts of resistance exercise. J. Sports Sci. Med.

